# The Utility of Neutrophil CD64 and Presepsin as Diagnostic, Prognostic, and Monitoring Biomarkers in Neonatal Sepsis

**DOI:** 10.1155/2020/8814892

**Published:** 2020-11-01

**Authors:** Heba E. Hashem, Rania M. Abdel Halim, Sherin A. El Masry, Amira M. Mokhtar, Noureldin M. Abdelaal

**Affiliations:** ^1^Clinical Pathology Department, Faculty of Medicine, Ain Shams University, Cairo, Egypt; ^2^Pediatric and Neonatology Department, Faculty of Medicine, Ain Shams University, Cairo, Egypt

## Abstract

**Background:**

Neonatal septicemia is a critical medical situation; current conventional laboratory methods still have many limitations and diagnostic obstacles. For this purpose, last decades have witnessed a challenge between the battery of sepsis biomarkers including many leukocyte surface antigens, not only for early diagnostic purposes but also for better follow-up and good management of sepsis patients.

**Aim:**

To evaluate the diagnostic, prognostic, and monitoring performance of both neutrophil CD64 (nCD64) and presepsin as sepsis biomarkers compared to each other and to the conventional laboratory sepsis parameters aiming to decide which is the best fitting for routine daily use in neonatal intensive care units (NICUs).

**Methods:**

235 neonates were enrolled from three Egyptian neonatal ICUs, during the period from November 2015 till March 2018; they were classified into two main groups: the control group (*n* = 102) and the sepsis group (*n* = 133). Laboratory sepsis evaluation included highly sensitive CRP (hs-CRP), CBC, in addition to nCD64 (flow cytometry technique), and presepsin measurement (CLEIA technique combined with Magtration® technology); the diagnosis was confirmed thereafter by positive blood culture results (BacT/Alert system). Sixty-two of the enrolled sepsis neonates were subjected to follow-up assessment; they were reclassified according to their clinical improvement at the second time assessment into (group 1: sepsis group without improvement) (*n* = 20) and (group 2: improved sepsis group) (*n* = 42).

**Results:**

Significant increase in nCD64 and presepsin values was found in sepsis groups compared to the controls. At cutoff 41.6%, nCD64% could discriminate the presence of septicemia with sensitivity 94.7%, specificity 93.6 %, and AUC 0.925, while presepsin at cutoff 686 pg/ml achieves sensitivity 82.7%, specificity 95.5%, and AUC 0.887, respectively. Significant increase in nCD64 (*P* < 0.001) and hs-CRP (*P*=0.018) values was observed in severe sepsis/septic shock patients compared to nonsevere sepsis patients. Delta change percentage (dC%) between initial and follow-up evaluations for both improved and nonimproved sepsis patients was dC *Z* value −5.904 for nCD64% followed by dC *Z* value −4.494 for presepsin.

**Conclusion:**

nCD64 and presepsin are valuable early diagnostic and monitoring sepsis biomarkers; the highest sensitivity could be achieved by a univariant sepsis marker in this study was recorded by the nCD64% biomarker, while the highest specificity was documented by presepsin. Combined measurement of both achieves the highest diagnostic performance in sepsis neonates. Either of CD64 or presepsin combined with hs-CRP associated with better performance than any of them alone. nCD64 carries an additional promising role in reflecting sepsis prognosis.

## 1. Introduction

Neonatal sepsis (NS) is an important cause of neonatal morbidities and mortalities worldwide [1, 2]; the overall incidence of NS ranges from 1 to 5 cases per 1,000 live births in developed countries, comparable to 49–170 per 1,000 live births in developing countries, with case fatality rates ranging from 2% to 60% [3, 4].

In Egypt, around 80% of early childhood deaths take place during the first year of life, with over half occurring during the first month of life. The neonatal mortality rates were documented as 14 deaths per 1,000 births [5].

Early diagnosis of sepsis is a clinical challenge as clinical symptoms and signs of the disease are subtle, late, and nonspecific making it hard to be discriminated from that of noninfectious causes [6]. Blood culture is still the gold standard for sepsis diagnosis, but minimum two days are required for the earliest result, in addition to the presence of many confounding factors giving false positive and false negative results which add another diagnostic obstacle [4, 7].

As a result, in the past decades, attention has been directed to different sepsis biomarkers other than the conventional parameters; these biomarkers include leukocyte cell surface antigens such as nCD64, CD14, CD11b, CD163, and soluble CD14 subtype (presepsin) [8, 9].

Neutrophil CD64 (nCD64) is one of the most researchable sepsis biomarkers; it shows a valuable role in both diagnosing and monitoring infections in both early-onset and late-onset sepsis in either term or preterm newborns [4, 6, 9–13]. Further studies postulated a valuable prognostic role for nCD64 and diagnostic efficacy to discriminate between infectious and noninfectious systemic inflammatory response syndrome (SIRS) in ICU settings [14, 15].

nCD64 is a type of integral membrane glycoprotein known as Fc receptor that binds monomeric IgG with high affinity [6]; it mediates many immunological responses including endocytosis, phagocytosis, cytokine release, and superoxide generation [16]. nCD64 is constitutively expressed on monocytes and macrophages; however, it is expressed at low concentration on nonactivated neutrophils but can be markedly increased at the onset of sepsis process [6, 17, 18].

Besides the valuable role of nCD64 in systemic infection and sepsis, there are a wide variety of diseases in which the diagnostic and prognostic utility of nCD64 has been proved; one of its most useful applications is to distinguish bacterial infections from the acute flares in rheumatoid arthritis and other autoimmune disorders [19]. Additionally, elevated nCD64 is proved to be a reliable prognostic biomarker for acute exacerbating chronic obstructive lung disease (COPD) [20], a sensitive and specific marker for early diagnosis of pneumonia and inflammatory bowel diseases, and a marker of systemic inflammation in HIV-infected patients [21, 22]. Furthermore, nCD64 has successfully been used as a rejection marker in transplant patients [23].

Soluble CD14 subtype (sCD14-ST) is another promising sepsis marker which is named as presepsin. It is a specific, high-affinity receptor for complexes of lipopolysaccharides and is involved in the recognition of a wide variety of bacterial products such as peptidoglycans; following its stimulation by pathogens, a soluble CD14 subtype is released in the circulation by shedding from the surface of the membranes of various immune cells, such as macrophages, monocytes, and neutrophils [4, 24], which can be measured by different immunological methods. Several studies suggest promising results for presepsin as an early diagnostic and prognostic sepsis marker [4, 9, 25–33].

Previous studies suggest a valuable role for each of nCD64 and presepsin in sepsis patients separately, but little of them compared the diagnostic and monitoring performance of both biomarkers together in the same sepsis evaluation, so our study aimed to investigate and compare the utility of nCD64 and presepsin, as early diagnostic, prognostic, and monitoring biomarkers in NS, individually and in combination to determine the most useful marker to be routinely applicable, and hence, proper timely management could be initiated and maintained.

## 2. Materials and Methods

### 2.1. Study Design

The current study was a prospective hospital-based case-control study carried out during the period from November 2015 till March 2018; subjected neonates were selected from three neonatal intensive care units (NICUs) (NICU of Obstetric and Gynecological Hospital, Ain Shams University, NICU of Pediatric Hospital, Ain Shams University, and NICU of Pediatric Department, Ain Shams University Specialized Hospital, Cairo, Egypt).

Written informed consent was received from the parents of the subjected neonates. The study was approved by the Research Ethics Committee of Ain Shams University Hospitals, Faculty of Medicine, and all procedures were in accordance with the Helsinki Declaration [34].

### 2.2. Sepsis Identification

Neonates were recruited in the study according to the new Ballard score [35], and they were admitted to Ain Shams University Hospital's NICUs.

For sepsis patient's identification and selection, neonates had presumed one or more infection risk factors [36], in addition to at least 2 clinical and 2 laboratory criteria: (1) respiratory compromise: respiratory rate >60 breaths per minute, or cessation of respiration for ≥20 seconds, occurring at a rate of ≥2 times per hour, or pulse oxymeter readings of ≤85%; (2) cardiovascular compromise: heart rate <100 beats per minute, pallor, or hypotension; (3) metabolic changes: hypothermia (rectal temperature <36°C), a body temperature of >38°C, feeding intolerance (increased gastric residuals >50% of milk volume in ≥2 feedings within 24 hours), glucose instability (blood glucose level <45 mg/dL or >125 mg/dL), or metabolic acidosis (pH < 7.25); or (4) neurologic changes: lethargy or decreased activity [37], whereas laboratory criteria were [38] white blood cell (WBC) count <5 or >20 × 10^9^/L, immature to total neutrophil (I : T) ratio >0.2, platelet count <100 × 10^9^/L, and CRP >10 mg/L. The diagnosis was verified thereafter by the gold standard for sepsis diagnosis, the positive blood culture.

### 2.3. Exclusion Criteria

Patients who had confirmed intrauterine viral infection (toxoplasmosis, rubella, cytomegalovirus, syphilis, and herpes), patients with long-standing hospitalization (admitted for more than one-month duration), and those neonates who had recently undergone surgical intervention were excluded from the study.

### 2.4. Group Classification

Subjected neonates were classified into two main groups: the control group and the sepsis group. The controls (group 1) were postnatal age- and sex-matched newborns, which included those neonates with no signs of infection (sepsis inclusion criteria were excluded in addition to negative CRP results).

The controls were subdivided into two subgroups: (group 1a) healthy controls, which included healthy neonates (cord blood samples) and (group 1b) diseased control group, which included those neonates with no signs of infection but subjected to sampling for performing investigations of different diseases including infants of diabetic mothers (IDM), premature neonates, neonatal physiological jaundice, hypoglycemic neonates, neonatal convulsions, Hirschsprung disease, and duodenal atresia.

The identified sepsis neonates (group 2) were further subdivided into two subgroups: (group 2a) documented sepsis patients, which included those neonates with the clinical diagnosis of sepsis plus positive blood culture and (group 2b) clinical sepsis patients, which included those neonates with the clinical diagnosis of sepsis but with negative blood culture results.

Sepsis patients were further reclassified according to the severity of the disease into the severe sepsis/septic shock group, which included those patients who fulfilled the clinical criteria of disease severity, and another group included nonsevere sepsis patients.

Besides the diagnostic and prognostic sepsis evaluations, sixty-two of the enrolled neonates were subjected to follow-up assessments. The second clinical and laboratory evaluations were performed on the 5th day from the baseline evaluation. According to the neonates' clinical condition, they were reclassified into two groups: (group 1: nonimproved sepsis patients) and (group 2: improved sepsis patients).

### 2.5. Patients' Evaluation and Laboratory Investigations

Newborns enrolled in the present study were subjected to the following:

Detailed medical history and thorough clinical examination.
Samples of peripheral blood for laboratory sepsis investigations; blood samples from the sepsis group were withdrawn as early as the neonate was clinically suspected to have sepsis signs and symptoms, and the laboratory sepsis profile included the following:
  - Complete blood count (CBC) (Coulter LH750 Analyzer, Beckman Coulter Inc., USA) and peripheral blood smears for the differential count.
  - Chemistry profile for both clinical judgment and follow-up purposes including alanine transaminase (ALT), aspartate transaminase (AST), blood urea, and serum creatinine (Cobas Integra 400 plus; Hoffman-La Roche Ltd., Basel, Switzerland).
  - Arterial blood gases (Cobas 221; Hoffman-La Roche Ltd.).
  - Highly sensitive CRP (hs-CRP) (Dimension® Clinical Chemistry System^∗^) (Siemens Healthcare Diagnostic Products GmbH, Emil-von-Behring-Str. 76, 35041 Malburg/Germany) using Flex® reagent cartridge.
  - Blood culture: 2 mL of blood was injected into the BacT/Alert culture bottle under complete aseptic conditions. The inoculated culture bottles were placed in the BacT/Alert instrument (bioMérieux, Marcy l'Etoile, France) as soon as possible, for incubation and monitoring. Positive samples were Gram-stained and subcultured on blood agar, MacConkey agar, and chocolate agar; then, plates were incubated in appropriate temperatures. Full identification of organisms was done with Vitek 2 Compact (bioMérieux).
  - Surface nCD64: flow cytometric analysis was performed on EDTA peripheral blood specimens obtained and analyzed within 48 hours of sample collection time; the test was conducted using the Leuko64 assay (Leuko64 kit, Trillium Diagnostics, Scarborough, ME, USA). Data analysis was performed using a Becton-Dickinson FACScan system. Compensation setting was established before acquiring the samples using color calibrated beads (BD Biosciences). After adjusting the sample count for acquisition, unstained samples were acquired to detect the sample autofluorescence. Isotype controls, mouse IgG1 fluorescein isothiocyanate (FITC) control, and mouse IgG PerCP control were obtained from BD Biosciences for the detection of nonspecific binding.
  - For each sample, 50 *μ*L of EDTA blood was stained by FITC-conjugated anti-human nCD64 (Immunotech, Beckman Coulter, Marseille, France) and peridinin-chlorophyll-protein complex- (PerCP-) conjugated anti-human CD45 (BD Biosciences). The optimal concentration was determined for each antibody by titration experiment.
  - After 15 minutes of incubation in the dark, each sample was washed, centrifuged, and lysed using BD FACS Lyse (BD Biosciences). Then, the samples were washed and resuspended in 200 *μL* FACS buffer (BD Biosciences).
  - Gating strategy: using CD45 and side scatter (CD45/SS), initial gating was performed on the neutrophil area in the dot plot graph for CD64. Data were expressed as mean fluorescence intensity (MFI) and percentage (%) using a single histogram, and each of the expression units was statistically analyzed.
  - Measurement of presepsin: 100 *μ*L of plasma samples were kept at −80°C until analysis. Presepsin was measured with the PATHFAST immunoanalyzer (PATHFAST®, Mitsubishi Chemical Medience Corp., Tokyo, Japan), a compact automated immune analyzer based on a noncompetitive chemiluminescent enzyme immunoassay (CLEIA) combined with Magtration technology [39, 40].

During incubation of the sample with the alkaline phosphatase-labeled anti-presepsin polyclonal antibody and anti-presepsin monoclonal antibody-coated magnetic particles, presepsin binds to the anti-presepsin antibodies forming an immunocomplex with the enzyme-labeled antibody and antibody-coated magnetic particles. After removing the unbound substances by Magtration technology, a chemiluminescent substrate is added. After a short incubation, the luminescence intensity generated by the enzyme reaction is measured. The luminescence intensity is related to the presepsin concentration of the sample which is calculated using a standard curve. The PATHFAST presepsin kit was used (LSI Chemical Medience Corporation., Japan) with an assay range of 20–20000 pg/ml.

All laboratory investigations were performed at the Clinical Pathology Departments, Ain Shams University Hospitals.

### 2.6. Statistical Analysis

Statistical analysis was performed by using SPSS statistical software package (IBM SPSS statistics V. 24.0, IBM Corp., USA, 2016). Data were expressed as median and interquartile range for quantitative nonparametric data and number, percentage for presenting qualitative data. The comparison between every two independent groups was done by the Wilcoxon rank-sum test, in addition to the correlation statistics (Spearman correlation) which was conducted for the possible associations between every two studied variables. The receiver operating characteristic (ROC) curve was used to assess the best cutoff point with sensitivity, specificity, positive predictive value (PPV), negative predictive value (NPV), +ve likelihood ratio (+LR), and −ve likelihood ratio (−LR) being calculated. The significance level was taken at *P* value ≤ 0.05. Additionally, for follow-up and monitoring purposes, delta change percentage (dC%) for each biomarker was calculated and compared.

Sample size was calculated using PASS 11.0 sample size calculation program. A total sample size of 235 neonates achieves 81% power to detect a change in sensitivity from 0.5 to 0.708 using a two-sided binomial test and 100% power to detect a change in specificity from 0.5 to 0.858 using a two-sided binomial test. The target significance level is 0.05. The actual significance level achieved by the sensitivity test is 0.0488 and achieved by the specificity test is 0.0415. The prevalence of the disease is 0.2.

## 3. Results

Two hundred and thirty-five neonates were included in the study, 107 males and 128 females with male to female ratio 1 : 1.2.

They were categorized into two main groups: control group (*n* = 102) and sepsis group (*n* = 133); control group was subdivided into (group 1a) healthy controls (*n* = 53) and (group 1b) diseased controls (*n* = 49), while sepsis patients were subdivided into two subgroups: (group 2a) documented sepsis patients (*n* = 65) and (group 2b) clinical sepsis patients (*n* = 68).

The comparison was conducted between sepsis versus the controls. Data were nonparametric, so the Wilcoxon rank-sum test was used. The demographic and clinical data of both groups are represented in Table 1.

### 3.1. Blood Culture Results

**Figure 1:**
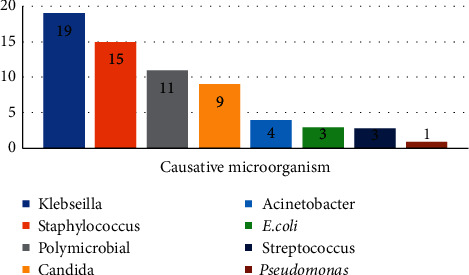
The distribution of the causative microorganisms.

Blood culture was positive in 48.8% of all septic neonates; *Klebsiella* was the most common microorganism isolated from NICU sepsis patients (*n* = 19, 14.28%) followed by coagulase-negative *Staphylococci* (CoNS) (*n* = 15, 11.27%) followed by more than monomicrobial infection (*n* = 11, 8.27%); other microorganisms are less commonly encountered in our NICUs including *Candida* spp. (*n* = 9, 6.76%), *Acinetobacter* (*n* = 4, 3%), *E. coli* (*n* = 3, 2.25%), *Streptococcus* spp. (*n* = 3, 2.25%), and *Pseudomonas* (*n* = 1, 0.75%) (Figure 1).

### 3.2. Outcome of Sepsis Patients

There was a highly significant increase in the number of deaths among sepsis patients compared to the controls (*P* < 0.001). By analyzing the outcome of sepsis patients after their follow-up until NICU discharge, 48/133 (36%) of them died from severe septicemia and its complications, while 80/133 (60%) were clinically cured and discharged and 5/133 (4%) were referred to other NICUs.

### 3.3. Laboratory Evaluation

Comparative statistical analysis between healthy controls, diseased controls, and sepsis patients regarding the studied laboratory parameters is illustrated in Table 2.

**Figure 2:**
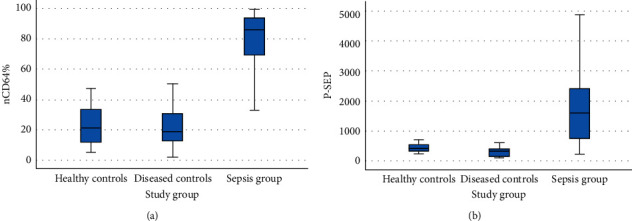
nCD64 and presepsin box plots for the healthy controls, diseased controls, and sepsis patients' groups. (a) nCD64% significantly increased (*P* < 0.001) in sepsis {median [IQR]: 85.6 (65.5–93.85)%} when compared to the healthy controls {median [IQR]: 21.3 (11.65–33.85)%} and the diseased controls {median [IQR]: 19 (12.85–31.25)%}, but no significant difference was found when healthy and diseased controls were compared together (*P*=0.934). (b) Presepsin significantly increased (*P* < 0.001) in sepsis {median [IQR]: 1645 (748–2425) pg./ml} when compared to the healthy controls {median [IQR]: 434 (339.5–595) pg./ml} and the diseased controls {median [IQR]: 339 (152.5–413) pg./ml}. Additionally, a statistically significant difference existed when healthy controls and pathological controls were compared together (*P*=0.001), being lower in the diseased controls than the healthy controls.

Figure 2 represents nCD64 and presepsin box plots in the healthy controls, diseased controls, and sepsis patients' groups.

Statistical analysis between the control group versus the documented sepsis patients was performed, and significant differences were documented between both groups by each of the following sepsis parameters: presepsin, nCD64%, nCD64 mean fluorescence intensity (nCD64 MFI), hs-CRP, platelet (PLT), Hemoglobin (Hb), and absolute lymphocyte count (ALC) (Table S1).

Table 3 demonstrates the comparative statistical analysis between documented and clinical sepsis subgroups as regards the laboratory parameters. nCD64%, nCD64 MFI, hs-CRP, PLT, Hb, and ALC achieved significant differences between both sepsis subgroups besides their significant differences between sepsis and control groups (Table 3).

Sepsis patients were further reclassified according to the severity of the disease into the severe sepsis/septic shock group (*n* = 37) which included those patients who fulfilled the clinical criteria of disease severity, and another group included nonsevere sepsis patients (*n* = 96); the comparison was conducted between both categories of patients, and the results are illustrated (Table 4).

No significant difference between early-onset sepsis and late-onset sepsis patients was found regarding nCD64 %, nCD64 MFI, hs-CRP, and presepsin (*P* > 0.05).

The diagnostic validity results for each marker and their combinations are summarized in Table 5.

**Figure 3:**
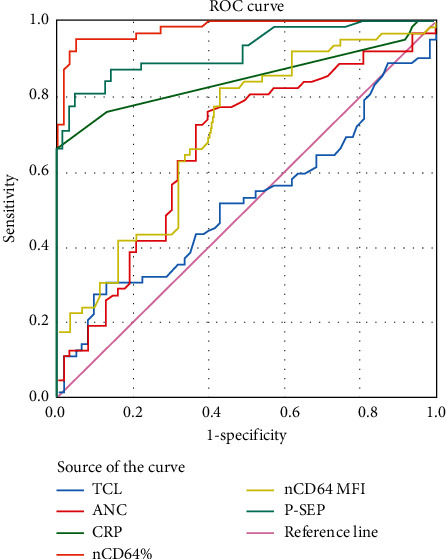
ROC curve analysis showing the diagnostic performance of studied biomarkers for the discrimination between sepsis patients and the control groups.

ROC curve for presepsin, nCD64, CRP, and CBC indices is illustrated in Figure 3, and the AUCs of the studied sepsis parameters were calculated (Table 5).

The multiregression analysis was constructed to identify the best panel of markers that can discriminate effectively between sepsis patients and the controls.

The first panel included all the studied sepsis parameters, from which *P* values were calculated for each; accordingly, the biomarker that gave a significant value was subjected for further analysis, and the *F*-ratio achieved by the first panel was 29.138 (Table S2). Another regression model was constructed, and the final one involved both (nCD64% and presepsin) which achieved the highest *F*-ratio that could be ever documented in the current study (Table 6).

Regarding the correlations existing between sepsis biomarkers, a highly significant negative correlation was documented between platelet count and each of the following: nCD64 % (rs = −0.24, *P*=0.007), nCD64 MFI (rs = −0.252, *P*=0.005), and hs-CRP (rs = −0.294, *P*=0.001), respectively. Additionally, nCD64 makes significant negative correlations with both hemoglobin (rs = −0.237, *P*=0.007) and absolute lymphocyte count (rs = −0.213, *P*=0.038), while significant positive correlation was documented between both nCD64 expression formulas (nCD64% and nCD64 MFI) (rs = 0.752, *P* ≤ 0.001).

Nonsignificant correlation was reported between hs-CRP with either nCD64% (rs = 0.141, *P*=0.111) or P-SEP (rs = −0.037, *P*=0.755).

### 3.4. Monitoring Performance of Studied Sepsis Biomarkers

Sixty-two of the enrolled neonates were subjected to further follow-up assessment after 5 days from the baseline evaluation. They were reclassified clinically into two groups: (group 1: nonimproved sepsis patients) (*n* = 20) and (group 2: improved sepsis patients) (*n* = 42).

The comparisons were conducted between the first baseline and the 2^nd^ monitoring evaluations for each patient in both groups. Regarding the improved sepsis group, significantly different values between both initial and follow-up evaluations were documented by each of the following sepsis parameters: nCD64%, P-SEP, hs-CRP, PLT, nCD64 MFI, ANC, and Hb (Table S3).

Concerning the nonimproved sepsis patients' group, all studied sepsis parameters show a nonsignificant difference (>0.05) between both evaluations suggesting their usefulness in the monitoring purpose of nonimproved sepsis neonates (Table S4).

### 3.5. Delta Change Percentage for Each Biomarker

Delta change percentage (dC%) for each biomarker was calculated (Table 7). The percentage change reflects how big the change was relative to the initial value, and it was calculated for each parameter by using the following equation:

(1) $$\begin{array}{c} \frac{\left[X\left(\text{final}\right)-X\left(\text{initial}\right)\right]}{X\left(\text{initial}\right)}*100, \end{array}$$

where *X* stands for the sepsis parameter result.

nCD64% followed by P-SEP, PLT, and hs-CRP could achieve promising results in the follow-up purposes of sepsis patients for both improved and nonimproved sepsis neonates.

## 4. Discussion

Septicemia is an obvious attributable cause to the high numbers of annual neonatal mortalities and morbidities, especially among developing countries [4, 41, 42]. Early diagnosis and proper management of NS are crucial for preventing long-term disabilities and life-threatening complications in addition to decreasing the emergence of antibiotic resistance and the financial burden, both at the levels of families and societies [4, 43].

Sepsis neonates were recruited in the current study (*n* = 133), while 102 neonates in whom signs and symptoms of infection were excluded were used as a control group. Nonsurvivor sepsis patients were representing 48/133 (36%) reflecting the severity and the comorbidity of NS among Egyptian neonatal ICUs.

The high rate of sepsis mortality is supported by the WHO and UNICEF which conducted updated systematic analysis for the neonatal and childhood mortality causes with time trends since 2000 till 2010; it is reported that sepsis accounts for 33% of total neonatal and childhood deaths [44]. Additionally, a prospective analytical study in three Egyptian NICUs in Mansoura Hospitals was conducted by El-Din et al. [45]; they reported the mortality rate from NS was very high which reached up to 50%.

Concerning the blood culture results, low diagnostic performance was documented in the current study as about half of subjected neonates with clinically suspected sepsis had negative blood culture results (51.2%). This came in concordance with Ng and Lam, who reported blood culture sensitivity in NS varies from 11% to 78% [46]. Also, a substantial group of studies supported its low diagnostic performance with sensitivities ranging from 25 to 45% of suspected cases [47, 48].

Regarding the causative microorganisms, *Klebsiella* was the commonest microorganism isolated from subjected sepsis neonates (*n* = 19, 14.28%) followed by coagulase-negative *Staphylococci* (CoNS) (*n* = 15, 11.27%). These results are in line with El-Madbouly et al. 2019 [4] who reported almost the same microorganism distribution.

In the present study, nCD64 (both nCD64% and nCD64 MFI) achieved significant differences between sepsis and each of the control groups being higher among sepsis neonates; it also achieved significant differences (*P*=0.002) between documented and clinical sepsis subgroups as well as between severe sepsis/septic shock patients and nonsevere sepsis cases. These valuable results in both diagnostic and prognostic aspects came in concordance with other study results [4, 6, 9, 49–52].

At cutoff value 41.6%, nCD64% achieved the highest sensitivity which could be documented by a univariant marker in the current study (94.7%), in addition to specificity 93.6%, PPV 95.5%, NPV 92.6%, efficacy 94.3%, and AUC 0.925, respectively. These results come in line with Morsy et al. [53] and Elkareem et al. [6] who reported nCD64 increased significantly in sepsis patients compared to the controls with comparable sensitivities, 95% and 96.1%, respectively.

On the contrary, results of a meta-analysis conducted by Shi et al. [54] indicated that nCD64 expression alone should be treated with caution in neonatal sepsis diagnosis, and they postulated that it is not a satisfactory marker for diagnosing neonatal sepsis.

Concerning presepsin's diagnostic validity results, besides, it has significant higher values in sepsis patients compared to each of the control groups. At cutoff value 686 pg/ml, presepsin achieved the highest specificity which could be documented by a univariant marker in this study (95.5%), in addition to sensitivity 82.7%, PPV 95.4%, NPV 83.1%, efficacy 88.7%, and AUC 0.887, respectively. These results come in line with several studies suggesting promising results for presepsin as an early diagnostic sepsis biomarker but with different cutoff values [4, 9, 25–33]. Moreover, they claimed that presepsin could be used as a useful marker to monitor treatment response as its levels decrease over time with treatment [55], which is also confirmed by the current study results.

Concerning the specificity of presepsin for sepsis diagnosis, besides its promising diagnostic validity results, its levels were higher in sepsis patients compared to the total controls, and even its values were lower in the diseased controls (median value = 339) than in healthy controls (median value = 434), which signifies the specificity of presepsin as a sepsis biomarker. Additionally, by comparing each of the control groups separately with the sepsis group, the results were higher in sepsis neonates rather than any of the controls.

On contrary to the present study, de Guadiana Romualdo et al. [56] reported that although presepsin is a valuable sepsis biomarker, its introduction in clinical practice for diagnosis of infection/sepsis is not justified.

Concerning hs-CRP, in the current study, a lower diagnostic performance was recorded by hs-CRP than that achieved by either nCD64% or presepsin. At cutoff value 6 mg/L, hs-CRP had sensitivity 71%, specificity 94.1%, NPV 71.6%, PPV 93.9%, efficacy 81.1%, and AUC 0.575, respectively.

The remarkable diagnostic performance of both nCD64 and presepsin in our study, comparable to the conventional sepsis parameters including hs-CRP, and CBC indices, suggests their potential utility as reliable markers for early diagnosis of NS; this was supported by other study results [4, 6, 9].

It was evident for us during the study course that both nCD64 and presepsin rise in the early stages of sepsis process, being earlier than CRP, and decrease more rapidly and dramatically with proper treatment and clinical improvement. This was confirmed by Gilfillan and Bhandari [9] who reported that presepsin rises almost 2–12 hours after the onset of infection, while nCD64 rises 1–6 hours after the sepsis onset; however, CRP rises later than both nCD64 and presepsin which rises 12–24 hours after infection and peaks at 36–48 hours and then drops dramatically with antibiotic treatment which makes CRP less than ideal for early sepsis diagnosis.

Concerning which of both (nCD64 or presepsin) is the best for sepsis diagnostic purposes, in the present study, nCD64 expression achieved a higher efficacy and sensitivity than presepsin which came in line with Stubljar and Skvarc [57] study results.

Supporting our results, by comparing the diagnostic validity of nCD64, presepsin, and CRP to decide which is the best of them for early diagnosis, several studies reported sensitivity 91% (95% CI 87%–93) for nCD64, 77%–80% for presepsin, and 49%–68% for CRP, respectively, while the reported specificity for the same biomarkers was 91% (95% CI 88%–94), 74%–83%, and >90%, respectively [9, 16, 54, 58, 59].

Additionally, Gilfillan and Bhandari [9] documented that serial measurement of nCD64 offers promise (better than presepsin) in the decision to initiate and/or control the duration of antibiotic therapy.

On the contrary, El-Madbouly et al. [4] conducted a comparative study between nCD64 and presepsin; they documented that both the sensitivity and the specificity of presepsin were superior to those achieved by nCD64 for early detection of NS, and they supported their results by other study results with similar statistical findings [60–62].

In the present study, multiregression analysis and statistical validity results documented that the combined measurement of nCD64% and presepsin recorded the highest results with sensitivity 98.6%, specificity 100%, PPV 100%, NPV 98.5%, and efficacy 99.3%, respectively. The second higher efficacy was achieved by a combination of nCD64% with hs-CRP, while hs-CRP in combination with presepsin came in the following ranking. Indeed, these results come in concordance with other studies [4, 37, 63, 64]. There is no doubt that these different biomarker combinations can improve their sensitivities and/or specificities, and this is generally more helpful when considered together because sepsis is a complex and dynamic syndrome.

Concerning the statistical correlations, a highly significant negative correlation (*P* < 0.001) between the platelet count and the parameters hs-CRP, nCD64%, and nCD64 MFI was observed. This negative correlation between nCD64% and the platelet count was in concordance with Mazary et al. results [65]. It is well known that thrombocytopenia is one of the most common complications of NS and is considered one of the hematological parameters reflecting sepsis severity [66]; this in turn could potentiate that nCD64 is a parameter of sepsis severity, too.

Besides the diagnostic sepsis evaluations, sixty-two of the enrolled neonates were subjected to follow-up assessments. Delta change percentage between nonimproved and improved sepsis neonates showed that each of the following biomarkers could achieve better results in sepsis follow-up purposes: nCD64% (dC *Z* value: −5.904) followed by presepsin (dC *Z* value: −4.494), platelet count (dC *Z* value: −2.899), and hs-CRP (dC *Z* value: −2.874).

The beneficial role of nCD64 in sepsis monitoring and follow-up purposes is supported by other studies; they reported a quick reduction in nCD64 values correlating with sepsis patients' clinical improvement [10, 16, 50, 52, 57, 67]. The second monitoring ranking was recorded by presepsin, which adds another advantage to its clinical application besides its diagnostic capability; this result was supported by other results [68–70].

The significant difference between severe sepsis/septic shock patients and nonsevere sepsis patients achieved by nCD64 makes it a more valuable biomarker over presepsin as a marker for sepsis severity, which was agreed by others [71, 72].

A substantial number of nonsevere sepsis patients in our study had a high level of presepsin at their initial evaluations that reached to the level of severe sepsis cases or even exceeding >2500 pg/ml. However, later on, upon monitoring, a dramatical rapid decline of presepsin was documented concordant with patients' clinical improvement. So, presepsin can be considered a valuable monitoring sepsis marker rather than being a marker of sepsis severity.

Concerning the technical aspects, nCD64 expression has shown particular advantage as its flow cytometric measurement is rapid (turnaround time takes maximum 2 hours), and it needs only a small blood volume to be performed (50 *μ*L of whole blood is sufficient) [73]. Moreover, the expression of nCD64 is not affected by transient tachypnea of the newborn, respiratory distress syndrome, and other noninfective perinatal events [4, 74]. In addition to its reported utility in both early-onset and late-onset sepsis, besides, the high sensitivities and specificities were documented when combined with the hematological indices and CRP [9, 68].

Concerning the nCD64 clinical application in the routine daily work as a point-of-care testing, Hassan et al. [18] demonstrated a robust design of a point-of-care microfluidic biochip for quantification of the nCD64 expression from whole blood that can potentially be used at the patient's bedside for continuous monitoring of sepsis patients in response to different therapeutic interventions at various stages of the disease.

A meta-analysis conducted by Jia et al. concluded that the nCD64 expression can be used as an additional test in the diagnosis of neonatal infection [75]. However, different cutoff values by using different expression units of nCD64 were reported in many studies with variable sensitivities and specificities, adding a diagnostic obstacle to its routine clinical application [65, 76].

Concerning the technical aspect of presepsin, a small sample volume is needed per test (100 *μ*L of plasma is sufficient), rapid turnaround (readily available as a point-of-care testing in ER settings); high sensitivities and specificities are recorded from previous studies. Moreover, cord blood levels have high predictive value (for early-onset sepsis in late-preterm and term infants) than other biomarkers [9]. However, the main disadvantages of its clinical application are the variation in cutoff values used between the studies besides the limited information on its role in clinical decision-making which adds another medical limitation [9, 76].

Comparing CRP as a standard sepsis biomarker being widely used in worldwide NICUs, it is available with reasonable turnaround time, high specificity, and lower financial cost comparable to the new biomarkers. Additionally, its levels fall dramatically with effective treatment. On the opposite side, CRP rises late in the time course of infection, so single measures early in the course of the illness are not reliable; moreover, its interpretation can be confounded by other physiological and pathological conditions including even the age of the neonate and the mode of delivery, which in fact make it less than ideal sepsis biomarker [9, 77].

Lastly, despite that either nCD64 or presepsin is more fitting for routine clinical application as a part of daily sepsis profile evaluation, both are more costly than CRP, but indeed, the high rate of neonatal mortality resulting from septicemia, in addition to the expected lifelong morbidities among the survivors, besides the increasing rates of antibiotic resistance and the huge financial burden, both at the levels of families and societies, makes the routine application of such early diagnostic and better monitoring sepsis biomarkers a critical and urgent need that should be taken in utmost considerations.

## 5. Conclusion

Neutrophil CD64 and presepsin biomarkers are significantly higher in sepsis patients than the controls, suggesting their potential use for early diagnosis of NS in routine clinical situations.

The highest sensitivity could be achieved by a univariant sepsis marker is recorded by the nCD64% biomarker, while the highest specificity was documented by presepsin. Despite hs-CRP achieved remarkable specificity, it is associated with undesirable sensitivity, making it less ideal in early sepsis diagnosis.

The best diagnostic efficacy for early sepsis diagnosis can be reached by combined measurement of both nCD64% and presepsin.

Either nCD64 or presepsin can be used individually, while the combined measurement of nCD64 or presepsin with hs-CRP gives a better diagnostic validity than any of them alone.

Both nCD64 and presepsin are valuable markers for monitoring purposes of neonatal septicemia; one should interpret the results of sepsis laboratory parameters together and in context with the patient medical condition for the best clinical judgment.

nCD64 carries a promising role over presepsin in reflecting sepsis severity; further studies are recommended to determine the prognostic role of presepsin and nCD64 compared to each other.

### 5.1. Limitations of the Study

Limitations of this study must be addressed. First, procalcitonin was not included in our comparative study; however, it is well known to be a powerful diagnostic and prognostic biomarker in neonatal septicemia for two main reasons; firstly, it would be difficult to be performed routinely in our hospital because of financial issues and limited resources. Secondly, several studies compared procalcitonin with either nCD64 or presepsin in neonatal sepsis diagnosis and monitoring purposes; their results revealed better performance of either nCD64/P-SEP over procalcitonin or even the same, so it was intended in the current study to compare both promising biomarkers, nCD64 and presepsin, together to decide which of them is the best.

Second, not all neonates with sepsis had positive culture results, and this is could be attributed to many factors including the required blood volume to be inoculated, which is very critical in neonatal ICU settings, in addition to the diagnostic sensitivity of the used blood culture which proved to be less than optimum. Furthermore, the etiological agent may not be isolated by media used in our study such as anaerobes, viral (e.g., rubella and cytomegalovirus), protozoal (e.g., *Toxoplasma gondii*), and treponemal (e.g., *Treponema pallidum*) pathogens.

Third, neonates with congenital malformations, chromosomal abnormalities, and surgical interventions were not excluded from the study; in addition, the control group included nonsepsis diseased neonates besides the healthy controls. Indeed, this was intended to test the clinical application of sepsis biomarkers in different heterogeneous groups of patients (which reflect the daily circumstances of our NICUs) before being routinely applicable.

## Figures and Tables

**Table 1:** Demographic and clinical data of the studied groups.

Parameter	Control group (*n* = 102)	Sepsis group (*n* = 133)	*P* value
Preterm (GA < 37 w)	31 (30.39%)	67 (50.37%)	<0.001
LBW and VLBW	33 (32.35%)	58 (43.6%)	0.136
Male gender	41 (40.19%)	63 (47.36%)	0.205
Vaginal delivery	41 (40%)	62 (47%)	0.205
Surgical intervention	3 (2.9%)	33 (25.4%)	<0.001
Respiratory support	5 (4.9%)	83 (62.4%)	<0.001
Rupture of membrane	2 (2.4%)	20 (15.03%)	<0.001
DOH	5.5 (1–35)	24 (3–127)	<0.001

Values are presented as number (%). *P*: probability value. GA: gestational age (weeks). LBW: low birth weight. VLBW: very low birth weight. DOH: duration of hospitalization.

**Table 2:** Comparative statistical analysis between sepsis and each of the control groups.

	Healthy controls	Diseased controls	Sepsis group	*P*1	*P*2
	Median (IQR)	Median (IQR)	Median (IQR)	Between healthy controls and sepsis patients	Between diseased controls and sepsis patients
Hb	16.95 (13.475–18.725)	12.6 (9.95–17.6)	12 (10.4–14.225)	<0.001	0.153
TLC	14.2 (11.675–17.225)	12.7 (8.95–17)	13.5 (9.15–19.725)	0.247	0.135
ANC	5.05 (3.475–10.85)	4 (2.7–9.6025)	7 (4.2–12.25)	0.472	0.003
ALC	5.6 (4.1–7.5)	5.7 (5–6.85)	4.3 (2.65-)7.15	0.186	0.009
AMC	1.4 (0.9–2)	1 (0.75–1.5)	1.45 (0.7–2.075)	0.098	0.092
PLT	213 (150–317)	347 (227.5–407)	200 (96.5–293)	0.667	<0.001
CRP	0.5 (0.5–0.6)	0.5 (0.5–0.5)	1.5 (0.6–4.8)	<0.001	<0.001
nCD64%	21.3 (11.65–33.85)	19 (12.85–31.25)	85.6 (65.5–93.85)	<0.001	<0.001
nCD64 MFI	1.5 (1.295–2)	1.42 (1.17–1.93)	1.86 (1.5–2.6)	0.015	<0.001
P-SEP	434 (339.5–595)	339 (152.5–413)	1645 (748–2425)	0.034	<0.001

Values are presented as median and IQR. Hb: hemoglobin (g/dl). TLC: total leukocytic count (×10^9^/L). ANC: absolute neutrophil count (× 10^9^/L). ALC: absolute lymphocyte count (× 10^9^/L). AMC: absolute monocyte count (×10^9^/L). PLT: platelet (×10^9^/L). hs-CRP: highly sensitive CRP (mg/L). nCD64%: neutrophil CD64%. nCD64 MFI: nCD64 mean fluorescence intensity. P-SEP: presepsin (pg/ml). P: probability value.

**Table 3:** Comparison between documented sepsis (group 2a) and clinical sepsis (group 2b).

	Documented sepsis group (group 2a)	Clinical sepsis group (group 2b)	*Z*	*P*
	Median (IQR)	Median (IQR)		
Hb	11 (9.7–12.2)	13.4 (11.5–15)	−5.078	<0.001
TLC	12.6 (9–16.5)	16.8 (9.3–22.7)	−2.106	0.035
ANC	6.85 (3.7–11.375)	7 (4.45–12.4)	−0.908	0.364
ALC	3.65 (2.175–5)	6 (3.4–8.6)	−3.43	0.001
AMC	1.2 (0.5–2)	1.61 (0.8–2.5)	−2.169	0.03
PLT	141 (63.5–218)	256 (167–344)	−4.304	<0.001
hs-CRP	24 (12–85.5)	10 (5–24)	−3.536	<0.001
nCD64 MFI	2.07 (1.62–2.945)	1.82 (1.415–2.405)	−2.428	0.015
nCD64%	88 (80.3–95.375)	80.7 (58.6–90.15)	−3.162	0.002
P.SEP	1645 (748–2373)	1571 (802.5–2683.75)	−0.085	0.932

Values are presented as median and IQR. Hb: hemoglobin (g/dl). TLC: total leukocytic count (×10^9^/L). ANC: absolute neutrophil count∗10 ^ 9/cmm^3^. ALC: absolute lymphocyte count (×10^9^/L). AMC: absolute monocyte count (×10^9^/L). PLT: platelet (cmm^3^). hs-CRP: highly sensitive CRP (mg/L). nCD64%: neutrophil CD64%. nCD64 MFI: nCD64 mean fluorescence intensity. P-SEP: presepsin (pg/ml). *P*: probability value. *Z*: Wilcoxon's rank-sum test.

**Table 4:** Comparison between severe sepsis/septic shock patients and nonsevere sepsis patients.

	Severe sepsis/septic shock group	Nonsevere sepsis patients	*Z*	*P*
	Median (IQR)	Median (IQR)		
Hb	11.15 (9.8–12)	13.1 (10.675–14.8)	−3.507	<0.001
TLC	10.65 (8.15–17.8)	14.6 (9.65–19.95)	−2.17	0.03
ANC	6.5 (2.4–12.4)	7 (4.4875–12.225)	−1.11	0.267
ALC	2.7 (1.225–4.35)	5 (3.4–8)	−3.853	<0.001
AMC	0.9 (0.4–2)	1.5 (0.8–2.325)	−2.517	0.012
PLT	90 (29–168)	240 (158–321.75)	−5.252	<0.001
hs-CRP	24 (9.75–96)	12 (6–37)	−2.368	0.018
nCD64%	94.1 (83–97.6)	81.85 (60.075–89.375)	−4.213	<0.001
nCD64 MFI	2.15 (1.76–3)	1.82 (1.44–2.41)	−2.958	0.003
P-SEP	1790 (766.25–2741.5)	1425 (739.5–2425)	−0.931	0.352

Hb: hemoglobin (g/dl). TLC: total leukocytic count (×10^9^/L). ANC: absolute neutrophil count (×10^9^/L). ALC: absolute lymphocyte count (×10^9^/L). AMC: absolute monocyte count (×10^9^/L). PLT: platelet (cmm3). hs-CRP: highly sensitive CRP (mg/L). nCD64%: neutrophil CD64%. nCD64 MFI: nCD64 mean fluorescence intensity. P-SEP: presepsin (pg/ml). *P*: probability value. *Z*: Wilcoxon's rank-sum test. No significant difference between early-onset sepsis and late-onset sepsis patients was found regarding nCD64%, nCD64 MFI, hs-CRP, and presepsin (*P* > 0.05).

**Table 5:** Diagnostic performance of the studied parameters and their combinations arranged in the ascending order in terms of their efficacy.

	Cutoff value	Sensitivity (%)	Specificity (%)	PPV (%)	NPV (%)	Eff. (%)	AUC	+ve LR	−ve LR
ANC	4.4	73.5	52.4	68.3	58.7	64.7	0.528	1.544	98.6
Hb	14.7	80.0	53.5	69.3	67.1	68.6	0.595	1.720	98.5
nCD64 MFI	1.43	80.8	53.2	70.5	66.7	69.2	0.574	1.726	98.5
hs-CRP	6	71.0	94.1	93.9	71.6	81.1	0.575	12.03	99.2
Presepsin	686	82.7	95.5	95.4	83.1	88.7	0.887	18.3	99.1
nCD64%	41.6	94.7	93.6	95.5	92.6	94.3	0.925	14.7	99
P-SEP and CRP	P-SEP at 686 pg/mL and CRP at 5 mg/L	95.9	100.0	100.0	95.7	97.9	0.977	NA	99.041
nCD64 and CRP	nCD64 at 41.6% and CRP at 6 mg/L	96.9	100.0	100.0	95.9	98.2	0.987	NA	99.031
Presepsin and nCD64	Presepsin at 245 pg/mL and nCD64 at 41.6%	98.6	100.0	100.0	98.5	99.3	0.997	NA	99.014

ANC: absolute neutrophil count (×10^9^/L). Hb: hemoglobin (g/dl). hs-CRP: highly sensitive CRP (mg/L). nCD64%: neutrophil CD64%. nCD64 MFI: nCD64 mean fluorescence intensity. P-SEP: presepsin (pg/ml). NA: not available.

**Table 6:** Multiregression analysis (final model).

Model 3	Reg. coef.	*t*	*P*	Sig.	*F*-ratio	*P*	Sig.
Item							
Constant	−0.14	−3.75	<0.001	HS			
nCD64%	0.012	16.741	<0.001	HS			
P-SEP	0.005585	3.535	0.001	HS			
					226.065	<0.001	HS

**Table 7:** Delta change percentage for both follow-up groups.

Biomarker	Improved sepsis group	Nonimproved sepsis group	*Z*	*P*
	Median (IQR)	Median (IQR)		
Hb.dC	−7.432 (−15.652–4.192)	−6.629 (−12.432–2.933)	−0.665	0.506
TLC.dC	−15.934 (−34.444–22.222)	−2.69 (−42.414–36.182)	−0.184	0.854
ANC.dC	−34.821 (−53.324–17.16)	20 (−28.571–50.602)	−1.986	0.047
ALC.dC	5 (−28.043–97.657)	−43.778 (− 84.285–8.201)	−2.05	0.04
AMC.dC	−32.143 (−64.744–27.083)	−37.5 (−66.611–17.704)	−0.134	0.893
PLT.dC	44.91 (0.295–163.462)	−2.41 (−72.24–42.045)	−2.899	0.004
hs-CRP.dC	−50 (−75–3.824)	100 (−50–300)	−2.874	0.004
nCD64%dC	−46.537 (−71.605–34.926)	5.734 (−3.855–60.157)	−5.904	<0.001
nCD64 MFI.dC	−26.337 (−51.262–8.698)	10.264 (−28.648–52.582)	−2.415	0.016
P-SEP.dC	−55.27 (−72.858–39.923)	−7.559 (−38.063–81.191)	−4.494	<0.001

## Data Availability

The data underlying the findings of this research are publicly available.

## Supplementary Materials

Supplementary Materials
*Description of the Additional Supplementary File*. Supplementary table (1): statistical comparison between the control group versus the documented sepsis patients; significant differences were observed between both groups by each of the following sepsis parameters: presepsin, nCD64%, nCD64 MFI, hs-CRP, PLT, Hb, and ALC. Supplementary table (2): multiregression analysis-first panel; all the studied sepsis parameters were included, from which *P* values were calculated for each, and the *F*-ratio achieved by this panel was 29.138. Supplementary table (3): a comparison between the first baseline and the 2^nd^ monitoring evaluations regarding the improved sepsis group; significantly different values were achieved by each of the following sepsis parameters: nCD64%, P-SEP, hs-CRP, PLT, nCD64 MFI, ANC, and Hb. Supplementary table (4): a comparison between the first baseline and the 2^nd^ monitoring evaluations regarding the nonimproved sepsis patients' group; all studied sepsis parameters showed a nonsignificant difference (>0.05) between both evaluations.
