# Supplementary material for: The Utility of Neutrophil CD64 and Presepsin as Diagnostic, Prognostic, and Monitoring Biomarkers in Neonatal Sepsis
Source: Int J Microbiol. 2020 Nov 1;2020:8814892. doi: 10.1155/2020/8814892 (PMC7654214; doi:10.1155/2020/8814892)
Supplement: Supplementary Materials — Description of the Additional Supplementary File. Supplementary table (1): statistical comparison between the control group versus the documented sepsis patients; significant differences were observed between both groups by each of the following sepsis parameters: presepsin, nCD64%, nCD64 MFI, hs-CRP, PLT, Hb, and ALC. Supplementary table (2): multiregression analysis-first panel; all the studied sepsis parameters were included, from which P values were calculated for each, and the F-ratio achieved by this panel was 29.138. Supplementary table (3): a comparison between the first baseline and the 2nd monitoring evaluations regarding the improved sepsis group; significantly different values were achieved by each of the following sepsis parameters: nCD64%, P-SEP, hs-CRP, PLT, nCD64 MFI, ANC, and Hb. Supplementary table (4): a comparison between the first baseline and the 2nd monitoring evaluations regarding the nonimproved sepsis patients' group; all studied sepsis parameters showed a nonsignificant difference (>0.05) between both evaluations. [file 8814892.f1.docx]

**Table (S1):** Comparison between control group and documented sepsis patients.

|  | **Control group** | **Documented sepsis group** | **Z** | **p** |
| --- | --- | --- | --- | --- |
| **Hb** | 14.8(11.3- 18.3) | 10.7(9.6-13.2) | -3.951 | <0.001 |
| **TLC** | 13.6(9.7- 17.2) | 13.5(10- 16.5) | -0.024 | 0.981 |
| **ANC** | 4.35(2.925- 10.375) | 7(5- 11) | -1.961 | 0.05 |
| **ALC** | 5.7(4.425- 7.25) | 4.75(3.075- 6.6) | -2.032 | 0.042 |
| **AMC** | 1.25(0.8- 1.9) | 1.4(0.8- 2.05) | -0.72 | 0.471 |
| **PLT** | 274(182.25-359) | 201(130- 264) | -2.148 | 0.032 |
| **Hs-CRP** | 0.5(0.5- 0.5) | 2.4(1.2- 4.8) | -8.002 | <0.001 |
| **CD64%** | 19.1(12.075- 32.125) | 83(78.3- 88) | -7.695 | <0.001 |
| **CD64 MFI** | 1.42(1.2375-1.9525) | 1.83(1.5- 2.7) | -2.797 | 0.005 |
| **P-SEP** | 340(245-500) | 1037(663- 1778.5) | -4.886 | <0.001 |

Values are presented as Median and (IQR)

Hb (hemoglobin) g/dl

TLC (total leukocytic count) * 10^9/ cmm3

ANC: Absolute neutrophil count * 10^9/ cmm3

ALC: Absolute lymphocyte count* 10^9/ cmm3

AMC; Absolute monocyte count * 10^9/ cmm3

PLT (Platelet) /cmm3

Hs-CRP (highly sensitive CRP) mg/L

nCD64% (neutrophil CD64%)

nCD64MFI (nCD64 mean fluorescence intensity)

P.SEP: Presepsin pg/ml

P, probability value

Z ^●^ = Wilcoxon's Rank Sum Test.

**Table (S2):** multi-regression analysis – model1

| Dependent Variable: Grps.sep | |  |  |  |  |  |
| --- | --- | --- | --- | --- | --- | --- |
| Model 1 |  |  |  |  |  |  |
| Item | Reg. Coef. | T | P | Sig. | F-Ratio | P |
| (Constant) | -0.317 | -0.374 | 0.709 | NS |  |  |
| GA | -0.012 | -1.141 | 0.257 | NS |  |  |
| BW | 0.00005852 | 1.541 | 0.127 | NS |  |  |
| Hb | 0.004 | 0.57 | 0.57 | NS |  |  |
| TLC | -0.012 | -1.377 | 0.172 | NS |  |  |
| ANC | 0.005 | 0.532 | 0.596 | NS |  |  |
| ALC | -0.009 | -0.864 | 0.39 | NS |  |  |
| AMC | 0.04 | 0.935 | 0.352 | NS |  |  |
| hs-CRP | 0.002 | 0.158 | 0.875 | NS |  |  |
| nCD64% | 0.011 | 9.411 | <0.001 | HS |  |  |
| nCD64.MFI | -0.011 | -0.445 | 0.657 | NS |  |  |
| P.SEP | 0 | 3.071 | 0.003 | HS |  |  |
|  |  |  |  |  | **29.138** | <0.001 |

**Reg. coef.:** regression coefient.

**Table (S3):** Comparison between baseline and follow up evaluations for improved sepsis groups.

| **Biomarkers**  **Improved group:** | **Initial level** | **Final level** |  |  |
| --- | --- | --- | --- | --- |
|  | **Median (IQR)** | **Median (IQR)** | **Z** | **p** |
| **Hb** | 12.7 (10.4 – 14.8) | 11.6(10.2 – 13) | -2.212 | 0.027 |
| **TLC** | 13.5 (9 – 18.2) | 13 (10- 15.3) | -1.535 | 0.125 |
| **ANC** | 6.08(4.55- 11.15) | 5.57 (3.725-7.075) | -2.783 | 0.005 |
| **ALC** | 4.1 (2.55-6.95) | 4.6(3.5-6.05) | -0.350 | 0.726 |
| **AMC** | 1.35(0.7-2.2) | 0.94 (0.5-1.2) | -1.859 | 0.063 |
| **PLT** | 197 (106 -319) | 283 (200 – 447) | -3.459 | 0.001 |
| **hs-CRP** | 23 (8 – 48) | 6 (5- 23.75) | -3.968 | <0.001 |
| **nCD64%** | 87.55 (74.5 - 96.375) | 35.05 (21.5 – 52.75) | -5.511 | <0.001 |
| **nCD64.MFI** | 2.37(1.48 - 3.145) | 1.45(1.21 -1.855) | -3.127 | 0.002 |
| **P.SEP** | 775(537.5 – 1244) | 275 (169.5 – 547) | -5.060 | <0.001 |

Initial level: first evaluation

final level: the 2^nd^ evaluation

Hb (hemoglobin) g/dl

TLC (total leukocytic count) * 10^9/ cmm3

ANC: Absolute neutrophil count * 10^9/ cmm3

ALC: Absolute lymphocyte count* 10^9/ cmm3

AMC; Absolute monocyte count * 10^9/ cmm3

PLT (Platelet) /cmm3

Hs-CRP (highly sensitive CRP) mg/L

nCD64% (neutrophil CD64%)

nCD64MFI (nCD64 mean fluorescence intensity)

P.SEP: Presepsin pg/ml

P, probability value

Z ^●^ = Wilcoxon's Rank Sum Test.

**Table (S4):** Comparison between the baseline and follow up evaluations for the non-improved sepsis group.

| **Biomarkers**  **(Continued**  **sepsis group)** | **Initial level** | **Final level** |  |  |
| --- | --- | --- | --- | --- |
|  | **Median (IQR)** | **Median (IQR)** | **Z** | **p** |
| **Hb** | 12.4(10.85- 14.025) | 11.15(10.3 – 13.8) | -1.349 | 0.177 |
| **TLC** | 14.7(9.475-18.975) | 14.3(9.125- 23.4) | -.411 | 0.681 |
| **ANC** | 6.5(3.9-9.1) | 9.2 (4.7 – 12.5) | -1.412 | 0.158 |
| **ALC** | 4.3(3.115-5.11) | 2.685(0.8175- 3.39) | -1.782 | 0.075 |
| **AMC** | 1.8 (1.1 – 2.6) | 1 (0.45 – 2.8) | -1.014 | 0.311 |
| **PLT** | 209(120 – 288) | 158(88- 234) | -1.046 | 0.295 |
| **hs-CRP** | 10.5(1.25 – 22) | 24(7.5 – 79.5) | -1.735 | 0.083 |
| **nCD64%** | 84.65(57.15 - 94.075) | 89(77 - 94.575) | -1.670 | 0.095 |
| **nCD64.MFI** | 2.115 (1.795 -3.155) | 2.69 (1.8125 - 3.4425) | -.806 | 0.42 |
| **P.SEP** | 1144(855.5 – 2504) | 3061(607- 4067.5) | -.674 | 0.5 |

Initial level: first evaluation

final level: the 2^nd^ evaluation

Hb (hemoglobin) g/dl

TLC (total leukocytic count) * 10^9/ cmm3

ANC: Absolute neutrophil count * 10^9/ cmm3

ALC: Absolute lymphocyte count* 10^9/ cmm3

AMC; Absolute monocyte count * 10^9/ cmm3

PLT (Platelet) /cmm3

Hs-CRP (highly sensitive CRP) mg/L

nCD64% (neutrophil CD64%)

nCD64MFI (nCD64 mean fluorescence intensity)

P.SEP: Presepsin pg/ml
